# HCG18, LEF1AS1 and lncCEACAM21 as biomarkers of disease severity in the peripheral blood mononuclear cells of COVID-19 patients

**DOI:** 10.1186/s12967-023-04497-6

**Published:** 2023-10-26

**Authors:** Simona Greco, Alisia Made’, Martina Mutoli, Lu Zhang, Santiago Nicolas Piella, Mélanie Vausort, Andrew I. Lumley, Antonio Paolo Beltrami, Prashant Kumar Srivastava, Valentina Milani, Sara Boveri, Marco Ranucci, Laura Valentina Renna, Hüseyin Firat, Antonino Bruno, Gaia Spinetti, Costanza Emanueli, Yvan Devaux, Fabio Martelli

**Affiliations:** 1 Molecular Cardiology Laboratory, IRCCS Policlinico San Donato, Via Morandi 30, 20097 San Donato Milanese, Milan Italy; 2 IRCCS MultiMedica, Via Fantoli 16/15, 20138 Milan, Italy; 3 Bioinformatics Platform, Data Integration and Analysis Unit, Luxembourg Institute of Health, 1445 Strassen, Luxembourg; 4 Cardiovascular Research Unit, Department of Precision Health, Luxembourg Institute of Health, 1445 Strassen, Luxembourg; 5 Department of Medicine (DAME), University of Udine, Udine, Italy; 6 National Heart and Lung Institute, Imperial College London, Hammersmith Campus, London, W12 0NN England, UK; 7 Laboratory of Biostatistics and Data Management, Scientific Directorate, IRCCS Policlinico San Donato, 20097 San Donato Milanese, Milan Italy; 8 Department of Cardiovascular Anesthesia and ICU, IRCCS Policlinico San Donato, Via Morandi 30, 20097 San Donato Milanese, Milan Italy; 9 Biobank BioCor, IRCCS-Policlinico San Donato, Via Morandi 30, 20097 San Donato Milanese, Milan Italy; 10 Firalis SA, 35 Rue du Fort, 68330 Huningue, France; 11 Laboratory of Immunology and General Pathology, Department of Biotechnology and Life Sciences, University of Insubria, Via Monte Generoso 71, 21100 Varese, Italy; 12 National Heart & Lung Institute, Imperial College London, Guy Scadding Building, Cale Street, London, SW3 6LY UK

## Abstract

**Background:**

Even after 3 years from SARS-CoV-2 identification, COVID-19 is still a persistent and dangerous global infectious disease. Significant improvements in our understanding of the disease pathophysiology have now been achieved. Nonetheless, reliable and accurate biomarkers for the early stratification of COVID-19 severity are still lacking. Long noncoding RNAs (LncRNAs) are ncRNAs longer than 200 nucleotides, regulating the transcription and translation of protein‐coding genes and they can be found in the peripheral blood, thus holding a promising biomarker potential. Specifically, peripheral blood mononuclear cells (PBMCs) have emerged as a source of indirect biomarkers mirroring the conditions of tissues: they include monocytes, B and T lymphocytes, and natural killer T cells (NKT), being highly informative for immune-related events.

**Methods:**

We profiled by RNA-Sequencing a panel of 2906 lncRNAs to investigate their modulation in PBMCs of a pilot group of COVID-19 patients, followed by qPCR validation in 111 hospitalized COVID-19 patients.

**Results:**

The levels of four lncRNAs were found to be decreased in association with COVID-19 mortality and disease severity: HLA Complex Group 18-242 and -244 (HCG18-242 and HCG18-244), Lymphoid Enhancer Binding Factor 1-antisense 1 (LEF1-AS1) and lncCEACAM21 (i.e. ENST00000601116.5, a lncRNA in the CEACAM21 locus). Interestingly, these deregulations were confirmed in an independent patient group of hospitalized patients and by the re-analysis of publicly available single-cell transcriptome datasets. The identified lncRNAs were expressed in all of the PBMC cell types and inversely correlated with the neutrophil/lymphocyte ratio (NLR), an inflammatory marker. In vitro, the expression of LEF1-AS1 and lncCEACAM21 was decreased upon THP-1 monocytes exposure to a relevant stimulus, hypoxia.

**Conclusion:**

The identified COVID-19-lncRNAs are proposed as potential innovative biomarkers of COVID-19 severity and mortality.

**Supplementary Information:**

The online version contains supplementary material available at 10.1186/s12967-023-04497-6.

## Introduction

COVID-19 is still a present and threatening global disease [1], which has reached over 700 million confirmed cases and almost 7 million deaths [2], 3 years after severe acute respiratory syndrome coronavirus 2 (SARS-CoV-2) identification. Consistent improvements in the knowledge of the disease’s pathophysiology have now been achieved. Nonetheless, reliable and accurate biomarkers for the stratification of COVID-19 severity in the initial phases of the disease are still missing. This fact poses the urgent need to understand the molecular basis and assess the clinical parameters underlying the disease severity, including the identification of laboratory indicators. Specifically, PBMCs have emerged as a source of indirect biomarkers reflecting the conditions of tissues; being composed of monocytes, B and T lymphocytes, and natural killer T cells (NKT) cells, they are highly informative for immune-related events [3, 4], a feature of particular interest in inflammatory diseases such as COVID-19.

LncRNAs are ncRNAs longer than 200 nucleotides, regulating the transcription and translation of protein‐coding gene expression [5–7]. Of relevance, lncRNAs can be found in the peripheral blood and hold a promising biomarker potential [8, 9]. Indeed, evidence is emerging that lncRNA expression in the PBMCs may be useful in COVID-19 patient stratification, although most of the studies published so far were conducted on a limited number of patients [10–13]. Moreover, a computational re-analysis of a publicly available PBMCs transcriptomics dataset performed on larger groups (n = 126 between COVID-19 patients and controls) [14] has been published and it identified several lncRNAs enriched in intensive care unit (ICU) COVID-19 patients [15], although no validation with an independent technique was reported.

In our study, we profiled the lncRNA expression pattern of a small subset of hospitalized COVID-19 patients taking advantage of the FIMICS panel [16]. This panel is enriched in cardiac-expressed lncRNAs, in keeping with the importance of cardiovascular complications for COVID-19 severity [17, 18], and it has been previously validated for its usefulness in the identification of disease biomarkers in the peripheral blood of patients affected by diseases with a strong inflammatory component, such as myocarditis and myocardial infarction [16]. Next, we went on validating the candidates in larger and independent patient groups, using independent techniques, identifying four lncRNAs transcripts differentially modulated in the PBMC from COVID-19 patients, displaying lower levels in critical or non-surviving patients.

## Methods

### Ethics approval and consent to participate

Studies were performed following the ethical principles according to the Helsinki Declaration of 1975 and as revised in 2013. The IRCCS Policlinico San Donato (PSD) experimental protocol was approved by the Institutional Ethics Committee of the San Raffaele Hospital (protocol number 75/INT/2020, 20/04/2020). The IRCCS MultiMedica (MM) experimental protocol was approved by the institutional review board ethics committee (protocol number 497.2021). All the patients enrolled in these studies were asked for their informed consent as previously approved by the ethics committee of each center.

### Patient enrollment

Patients were all positive for SARS-CoV-2 by qPCR assay and hospitalized either at PSD (Table 1, Additional file 1: Tables S1 and S2) or at MM (Additional file 1: Table S6). Anagraphic and clinical data were collected at hospital admission and patients were categorized according to the most severe COVID-19 grade observed during hospitalization as: (1) patients not requiring oxygen therapy, (2) patients requiring oxygen therapy, (3) patients requiring continuous positive airway pressure (CPAP) therapy and (4) patients admitted to intensive care unit and intubated (ICU) (Table 1, Additional file 1: Tables S1, S2 and S5).

**Table 1:** COVID-19 PSD patient’s characteristics, validation group

Characteristics_COVID-19 patients (n = 111)	Non-survivor COVID-19	Survivor COVID-19	Critical COVID-19 (class 4 and/or non-surviving)	Severe COVID-19 (alive and class 1 or class 2 or class 3)
Numerosity (n)	38	73	54	57
COVID-19 severity class (n)				
1 = O2 therapy not required	1	8	1	8
2 = O2 therapy required	2	29	2	29
3 = CPAP required in ward	8	20	6	20
4 = Intubation required in intensive care	24	16	45	0
Age (years, median ± SD)	**70.3 ± 10.4**	**64.5 ± 11.7**	67.5 ± 10.4	65.6 ± 12.6
Sex (n)	Male = 29 Female = 6	Male = 46 Female = 27	Male = 40 Female = 14	Male = 35 Female = 22
Smoke (n)				
Current or previous smoker	3	11	6	8
Non-smoker	35	62	49	48
Patient’s characteristics at hospital admission				
Body mass index (Kg/m^2^,median ± SD)	**26.0 ± 3.4**	**28.7 ± 6.2**	27.4 ± 3.7	28.1 ± 7.6
Hemoglobin (g/dl, median ± SD)	11.5 ± 2.2	11.9 ± 2.2	11.4 ± 2.1	12.1 ± 2.2
Hematocrit (%, median ± SD)	35.0 ± 6.5	36.6 ± 6.1	34.8 ± 6.2	37.3 ± 6.1
Red blood cells (10^6^/µl, median ± SD)	4.0 ± 0.7	4.1 ± 0.9	4.0 ± 0.7	4.2 ± 0.9
White blood cells (10^3^/µl, median ± SD)	10.2 ± 3.7	8.8 ± 5.1	**10.4 ± 5.3**	**8.3 ± 3.8**
Neutrophils (10^3^/µl, median ± SD)	9.4 ± 3.3	6.6 ± 4.6	**9.3 ± 5.6**	**6.1 ± 3.5**
Lymphocytes (10^3^/µl, median ± SD)	**0.6 ± 0.4**	**1.2 ± 0.7**	**0.8 ± 0.6**	**1.3 ± 0.7**
Monocytes (10^3^/µl, median ± SD)	0.7 ± 0.9	0.7 ± 0.7	0.8 ± 1.1	1.3 ± 0.7
Eosinophils (10^3^/µl, median ± SD)	0.02 ± 0.03	0.07 ± 0.02	0.07 ± 0.02	0.06 ± 0.12
Basophils (10^3^/µl, median ± SD)	0.01 ± 0.01	0.02 ± 0.05	0.03 ± 0.07	0.02 ± 0.01
Platelets (10^3^/µl, median ± SD)	222.4 ± 97.5	262.3 ± 117.6	226.5 ± 108.8	270.5 ± 112.5
Creatinine (mg/dl, median ± SD)	1.1 ± 0.8	1.0 ± 0.6	1.0 ± 0.8	0.9 ± 0.4
Reactive Protein C (mg/dl, median ± SD)	**11.6 ± 7.9**	**7.1 ± 8.2**	10.0 ± 9.3	7.3 ± 7.3
D-dimer (µg/mL, median ± SD)	**3.9 ± 4.6**	**1.4 ± 1.4**	**3.1 ± 4.0**	**1.4 ± 1.5**
Fibrinogen (mg/dl, median ± SD)	595.8 ± 151.0	564.3 ± 184.0	582.4 ± 163.9	567.8 ± 183.4
Comorbidities (n)				
Hypertension	24	39	32	31
Diabetes Mellitus	5	17	11	11
Obesity	6	15	13	8

Clinical parameters statistically different between non-surviving and surviving COVID-19 patients are reported in bold

The study conducted at PSD included 111 hospitalized patients aged 18 years or older, recruited during the period from March 2020 to January 2021, corresponding to the first and second COVID-19 waves, and 15 healthy controls (65.0 ± 3.8 years old, range 53.0–67.0 years, 1 female and 14 males). Peripheral blood mononuclear cells (PBMCs) and platelet-poor plasma samples from patients recruited at PSD were isolated by PSD BioCor Biobank at admission according to internal Standard Operating Procedures.

The study conducted at MM included 60 patients hospitalized for COVID-19 and consecutively enrolled from March to May 2021, corresponding to the third COVID-19 wave. PBMCs were isolated from the peripheral blood using the same Standard Operating Procedures used at PSD.

### PBMC and plasma sample collection and RNA isolation

PBMCs were isolated from 6 ml of peripheral blood in ethylenediaminetetraacetic acid dipotassium salt dihydrate (K_2_-EDTA) anticoagulant tubes by density gradient centrifugation at 1200×*g* for 10 min at room temperature with brake using Ficoll Histopaque Plus (Cytiva Sweden AB, cat# 17144003, lot #10308253) and SepMate-15 tube (STEMCELL Technologies, Canada, cat# 85415, lot#327374). Isolation of total RNA from PBMCs was performed using TRIzol RNA Isolation Reagent (Life Technologies, US, cat# 15596018, lot#19698001) and RNA concentration and purity were evaluated by NanoDrop One (Thermo Fisher Scientific Inc., US). Platelet-poor plasma was collected in K_2_-EDTA-anticoagulant tubes (BD Vacutainer, cat# 368860, lot# 2203608) and isolated as previously reported [19]. Briefly, cell- and platelet-free plasma was prepared following a 2-steps centrifugation protocol: samples were initially centrifuged at 1500*g* for 15ʹ at 4 °C. The supernatant was collected and centrifuged again at 14,000*g* for 15ʹ at 4 °C and plasma aliquots were stored at − 80 °C. RNA was isolated using NucleoSpin miRNA Plasma (cat# 740981.50, lot# 2105/001, Macherey–Nagel, Germany) according to the manufacturer’s instructions.

### RNA sequencing and data analysis

#### Library preparation

The Kapa biosystems’ KAPA Stranded RNA-Seq Kit with RiboErase (HMR) (cat#KK8484, Roche Diagnostics Corporation, US) was used to convert 500 ng of total RNA into a library of template molecules of known strand origin. The rRNA cytoplasmic component was removed by ribosomal depletion. Ribodepleted RNA was cleaned up using the Agencourt AMPure XP beads (cat#A63880, Beckman Coulter, Life Sciences, US) and DNAse I (cat#79254, Qiagen, Germany) digestion was performed. After these steps, the first and the second strands of cDNA were synthesized and an A-tailing reaction was performed for Illumina adapters ligation. DNA fragments were enriched by PCR and quality was checked on the Agilent Technologies 2100 Bioanalyzer (cat#5067-1504, Agilent, US) using a DNA 1000 chip according to the manufacturer’s recommendations.

#### FIMICS capture and sequencing

LncRNA quantification in PBMC samples was performed by using the FIMICS panel 2.0 which employs more than 55,000 probes of 120 nucleotides interrogating 2,906 lncRNAs [16]. This panel uses Celemics (Seoul, Korea) beads-based hybridization capture technology. In particular, biotinylated target capture probes were hybridized to the libraries at 65 °C for 24 h, then, captured lncRNA sequences were purified on T1 streptavidin-coated magnetic beads, and captured sequences were enriched by PCR (14 cycles). After PCR products purification with Beckman Coulter™ Agencourt AMPure XP beads (cat#A63880, Beckman Coulter, Life Sciences, US), the prepared libraries were sequenced by Illumina NextSeq 500 according to the manufacturer’s recommendation and a 100 bp paired-ends reads (2 × 100 bp) were generated.

#### Read mapping and quantification

Data were imported in the Partek Flow (flow version) 10.0.21.1015. The raw FASTQ files were trimmed at the 3’ end in function of their quality score (Phred score). The raw reads were aligned to the Homo sapiens hg19 reference genome using the software STAR v 2.7.3a [20] and default parameters were used. Then, mapped reads were quantified against the annotation with all lncRNAs from FIMICS panel with the Partek Expectation/Maximization (E/M) algorithm. Differential expression analysis was performed using the Limma R package [21] with quantile normalization. 2 CPM in at least half of the samples was used as the detection threshold.

### Analysis of single-cell transcriptomics datasets

Count matrixes and annotations were downloaded from the data resource of the studies describing single-cell RNA-sequencing from the blood [22], as well as heart and lung [23] of COVID-19 infected patients. The count matrix was processed using Seurat R package [24]. We combined the counts from the cells belonging to respective cell types (pseudo-bulk). The pseudo-counts per cell type per donor were further normalized into counts per million (CPM) values by edgeR R package and then the expression values were extracted.

### RT-qPCR

RNA was retro-transcribed using the GoScript Reverse Transcription kit (cat#A5001, lot# 0000578687, Promega Corporation, US) using 200 ng of total RNA according to the manufacturer’s instructions. Additionally, all RNA samples from MM were DNase I-digested (cat#79254, lot# 169040503 and 172015016, Promega Corporation, US). cDNAs were analyzed using the GoTaq qPCR Master Mix (cat#A6001, lot# 0000569038, Promega Corporation, US) according to the manufacturer’s instructions on a StepOne Plus instrument (Thermo Fisher Scientific Inc., US). Sequences of the adopted primers are reported in Additional file 1: Table S4. After normalization for UBC expression levels (PBMC) or 18S (plasma), relative RNA expression was calculated using the 2^^−ΔΔCt^ method [25]. The HCG-18, LEF1-AS1 and lncCEACAM21 PCR amplification products were characterized by the assessment of the dissociation properties of the double-stranded DNA by melting curves and by the measurement of the fragments size using 2.0% agarose gel (Additional file 2: Figure S1) followed by Sanger’s sequencing (data not shown).

### FACS analysis

PBMCs and neutrophils were isolated from the peripheral blood (20 and 12 mL respectively, in K_2_-EDTA anticoagulant tubes) collected at MM from healthy volunteers by density gradient centrifugation at 1200 rpm using Ficoll Histopaque®-1077 (Millipore Sigma, US, cat# 10771-500ML, lot# D8537-500 ML). The white ring interface, composed of PBMNCs, was used for T-lymphocytes, B-lymphocytes, monocytes, and NKT cell isolation by FACS-sorting. The bottom layer, composed of granulocytes (mostly neutrophils) and erythrocytes, following the depletion of erythrocytes, was used for neutrophil quantification by multicolor flow cytometry. Briefly: neutrophils (n = 14) were purified by lysis of erythrocytes, using ACK (Ammonium-Chloride-Potassium) Lysing Buffer. Neutrophils (10^5^) were stained using 2 µl/10^6^ cells (1:100 dilution) for 30 min at 4 °C with the following monoclonal anti-human antibodies (mAbs): FITC-conjugated CD45 (REA747, cat# 130-110-631, lot# 5211100512), PE-conjugated CD15 (VIMC6, cat#130-114-011, lot# 5211109970), APC-conjugated CD66b (REA306, cat#130-117-804, lot# 5211109949) and PerCP-Vio 700 conjugated CD14 (REA599, cat#130-110-581, lot# 521109954), all purchased from Miltenyi Biotec (Germany). Following morphological gating on viable cells and singlets selection, neutrophil enrichment was verified by CD45+/CD14−/CD15+/CD66b+ surface antigen expression, assessed by flow cytometry, using BD LSR FortessaTM X-20 Cell Analyzer. 2 µl/10^6^ cells (1:100 dilution) of the following mAbs were used for FACS Sorting of Monocytes, T- and B-lymphocytes (n = 8), and NKT cell (n = 6): FITC-conjugated CD45 (REA747, cat# 130-110-631, lot# 5211100512), APC-conjugated CD14 (REA599, cat# 130-110-520, lot# 5220305867), PerCP conjugated CD3 (BW264/56, cat# 130-113-131, lot# 5211008449), PE-conjugated CD19 (REA675, # 130-116-646, lot 5211108388) and APC-conjugated CD56 (REA196, cat# 130-113-310, lot# 5230100884). PBMNCs subpopulations were isolated by FACS-sorting using BD FACS-AriaII instrument as follows: CD45+/CD14−/CD19−/CD3+ (T-lymphocytes), CD45+/CD14−/CD3−/CD19+ (B-lymphocytes), CD45+/CD14+/CD3−/CD19− (monocytes) and CD3−/CD56+ (NKT). Gating strategies are shown in Additional file 3: Figure S2.

### Cell cultures and hypoxia experiments

Human T lymphoblast cell lines (THP-1) (ATCC, cat#TIB-202) and Jurkat (Clone E6-1, ATCC, cat#TIB-152) were maintained in RPMI 1640 medium (cat#BE12-115F/U1, Lonza, Westburg, The Netherlands) containing 10% FBS (cat#S181H-500, lot# S17193S181H, Biowest, US), 2 mM l-glutamine (cat# BE17-605E, Lonza, Westburg, The Netherlands), 1 × non-essential amino acids (cat# BE13-114E, Lonza, Westburg, The Netherlands) and 100 units of Potassium Penicillin and 100 μg of Streptomycin Sulfate/ml of culture media (cat# DE17-702E, Lonza, Westburg, The Netherlands).

For hypoxia experiments, THP-1 cells were incubated at 37 °C in a Ruskinn hypoxia cabinet (Baker, US) with 1% O_2_ and 5% CO_2_ and compared to cells incubated in normoxic conditions (37 °C; 21% O_2_; 5% CO_2_).

### Nuclear/cytoplasm fractionation

Nuclear/cytoplasm fractionation was performed in Jurkat cells using Paris Kit (cat#AM1921, Thermo Fisher Scientific Inc., US) according to the manufacturer’s recommendation. RNA was extracted using miRNeasy Micro kit (cat# 217084, Qiagen, Germany) and analyzed by RT-qPCR using Superscript™ II Reverse Transcriptase (cat#18064071, Thermo Fisher Scientific Inc., US) for cDNA synthesis and IQ SYBR Green supermix (cat#1708885, Bio-Rad, US) for real-time PCR on a CFX96 Real-Time PCR instrument (Bio-Rad, US).

### Statistical analysis

Continuous variables were expressed as mean ± standard error of the mean (SEM). For group-wise comparisons, two-tailed Mann–Whitney test or unpaired *t*-test were used as required. ANOVA test followed by Tukey’s post-hoc test was used to compare the means of more than 2 groups. All tests were performed 2-sided and a p < 0.05 was considered as statistically significant.

Descriptive statistics are expressed with counts and percentages for categorical variables and mean ± SD or median values with the interquartile range (IQR) for continuous variables.

A logistic regression model was used to evaluate adjusted odds ratios for age and sex. Receiver operating characteristic (ROC) curve, area under the curve (AUC) and odds ratio (OR) of each lncRNA related to the death or COVID-19 severity with and without the adjustment of age or sex were then calculated.

Statistical analyses were performed with SAS software, version 9.4 (SAS Institute, Inc., Cary, NC, USA) or with GraphPad Prism v.7.01 software (GraphPad Software Inc.).

## Results

### Identification of lncRNA candidates expressed in non-surviving COVID-19 patients

**Fig. 1:**
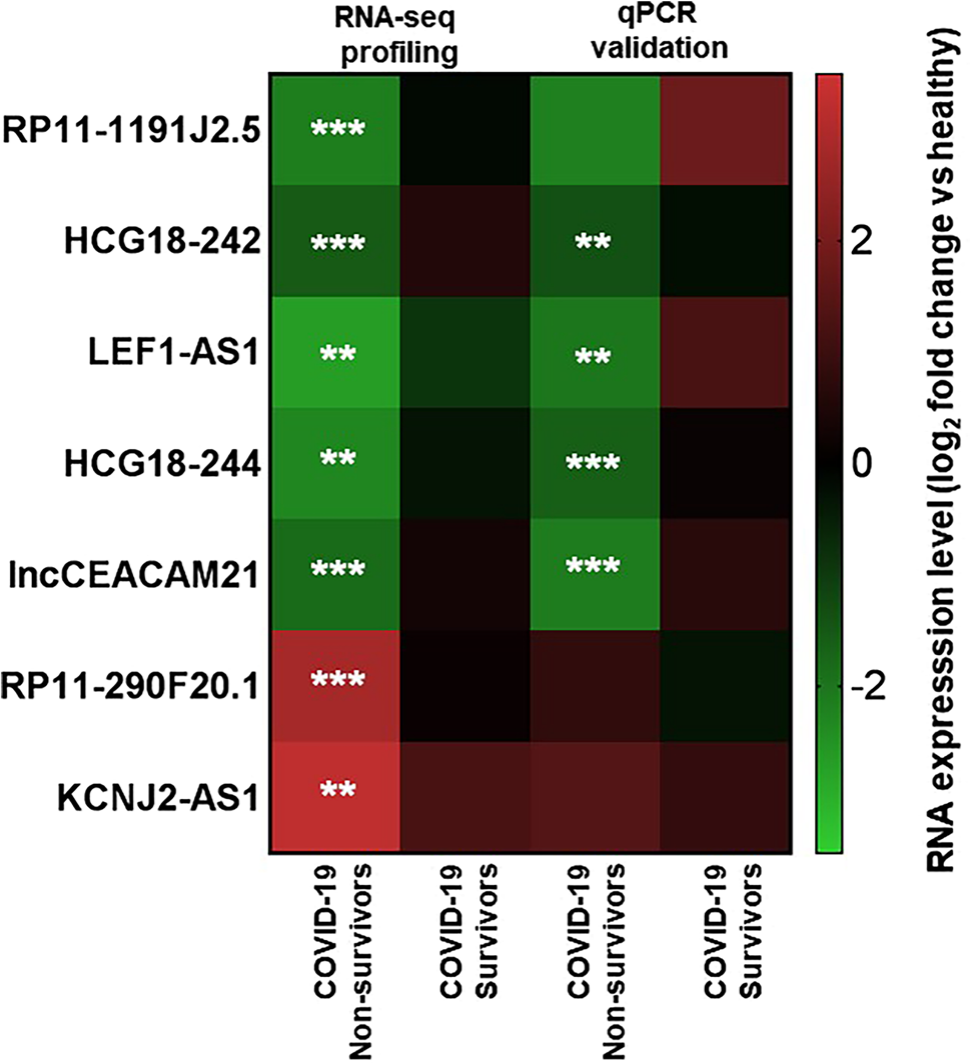
qPCR validation of RNA-Seq PBMC profiling data. Seven DE lncRNAs were identified for RT-qPCR technical validation of RNA-Seq profiling results. Heat-map shows RNA expression results in PBMC of 11 non-surviving and 21 surviving COVID-19 patients. Data are expressed as log_2_ fold change in non-surviving and surviving COVID-19 patients compared to healthy subjects. White asterisks indicate statistical significance for non-surviving compared to surviving patients (Mann–Whitney t-test; **p < 0.01, ***p < 0.001)

In a discovery phase, PBMCs were collected at admission at IRCCS Policlinico San Donato (PSD) hospital from 13 surviving and 12 non-surviving COVID-19 patients (Additional file 1: Table S1), matched for age and sex with 15 healthy controls. Total RNA was extracted and analyzed by the FIMICS probe panel, selecting 2906 cardiac-expressed lncRNAs, followed by RNA sequencing [16]. The rationale for the evaluation of the expression of cardiac lncRNAs in PBMC from COVID-19 patients derives from the reported shared comorbidities of cardiovascular diseases (CVDs) and COVID-19, and the finding of worse outcomes of COVID-19 associated with pre-existing CVDs conditions [26, 27]. 1,304 lncRNAs were detected and differential expression (DE) analysis identified 94 down-regulated (p < 0.05, fold change < − 1.0) and 51 up-regulated (p < 0.05, fold change >+ 1.0) lncRNAs by comparing surviving and non-surviving COVID-19 patients (Additional file 4: Figure S3). Logistic regression model analysis against survival was performed to calculate the odds ratio (OR) and the AUC (Area Under the receiver operator characteristic Curve). This analysis identified 21 lncRNAs that displayed both of the following features: (1) significantly related to survival (OR p < 0.05) and displaying an AUC > +0.8, both in a univariate logistic regression model and after the adjustment for age; (2) significantly differentially expressed in non-surviving compared to surviving COVID-19 patients (p < 0.05) (Additional file 1: Table S3). Seven lncRNAs of the 21 DE lncRNAs and significantly predicted to be associated with death were identified for RT-qPCR technical validation of the RNA-seq profiling based on the known/predicted biological functions of the lncRNA itself or of its neighbor coding gene, as well as according to technical parameters (e.g. the possibility to generate a specific qPCR assay) (Additional file 1: Table S4, highlighted in grey). Their expression levels were measured in total RNAs extracted from PBMC of 18 surviving and 14 non-surviving COVID-19 PSD patients, including also the patients used for the profiling step, as well as 15 healthy controls (Additional file 1: Table S2 and Fig. 1). Four lncRNAs, in particular HLA Complex Group 18, isoforms-242 and -244 (HCG18-242 and HCG18-244), Lymphoid Enhancer Binding Factor 1-antisense 1 (LEF1-AS1, ENST00000506314 LEF1-AS1) and lncCEACAM21 (a manually annotated lncRNA in the CEACAM21 locus, ENST00000601116.5), showed a statistically significant down-modulation in non-surviving patients, similar to that found by RNA-sequencing profiling (Fig. 1). The validated lncRNAs were collectively named COVID19-lncRNAs; the transcripts coordinate according to the GRCh38/hg38 genome annotation and the sequences of the primers are reported in Additional file 1: Table S4; figures in Additional file 1: Table S4 show GRCh38/hg38 UCSC Genome Browser sequence alignment of the HCG18, LEF1-AS1 and lncCEACAM21 transcripts.

### Validation of candidate COVID19-lncRNAs in a larger group of COVID-19 patients

The expression level of the COVID19-lncRNAs was measured by RT-qPCR in a larger group of PSD hospitalized COVID-19 patients, including some of the patients used for discovery profiling and technical validation (Table 1). The clinical characteristics of COVID-19 patients in the discovery profiling, technical and final validations did not differ significantly. COVID-19 patients were further classified according to the severity of the disease into critical (class 4 requiring ICU or non-survivors) and severe (classes 3 and 2 requiring CPAP or other oxygen therapy, respectively, class 1 not requiring oxygen therapy, without a lethal outcome) (Table 1 and Additional file 1: Tables S1 and S2). As expected, D-dimer levels were increased in non-surviving and critical patients, in line with consensus literature identifying D-dimer as a marker of activated coagulation and as a predictor for severity [28, 29].

**Fig. 2:**
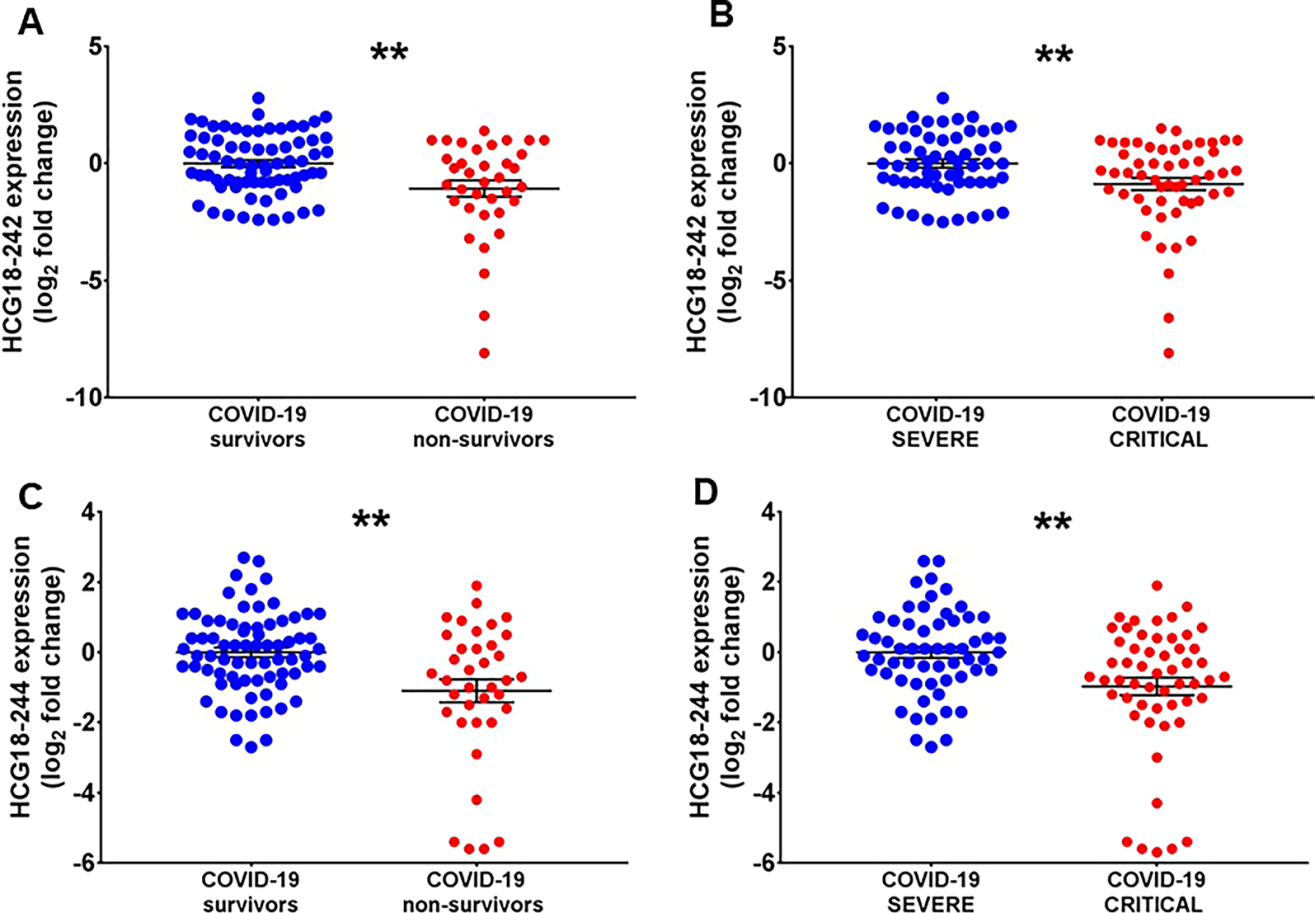
Decreased HCG18 short isoforms expression according to COVID-19 mortality and severity in COVID-19 patients recruited at PSD. Total RNA was extracted from PBMC derived from non-surviving (n = 35) and surviving (n = 73), or from critical (n = 55) and severe (n = 56) COVID-19 patients. Dot-plots show lncRNAs relative expression measured by RT-qPCR and expressed as log_2_ fold change. Mean values and standard error bars are indicated. Mann–Whitney t-test: ** p < 0.01. (**A**–**D**) show HCG18-242 and HCG18-244 data results, respectively

The expression levels of the COVID19-lncRNAs were compared between non-surviving and surviving patients, and also between critical and severe patients. Figure 2 shows that HCG18-242 and -244 transcripts levels significantly decreased according to mortality and also with disease severity. There are several HCG18 lncRNA transcripts annotated: HCG18-242 and -244 transcripts are formed by one exon only, but there are also several multi-exonic longer transcripts (Additional file 1: Table S4). To evaluate the modulation of longer HCG18 transcripts, we designed primers interrogating the sequence from exon 2 (ENSE00001767107) to exon 4 (ENSE00003882534) (Additional file 1: Table S4). We found that also the expression levels of longer HCG18 transcripts were significantly down-modulated in non-surviving and critical COVID-19 patients (Additional file 5: Figure S4).

**Fig. 3:**
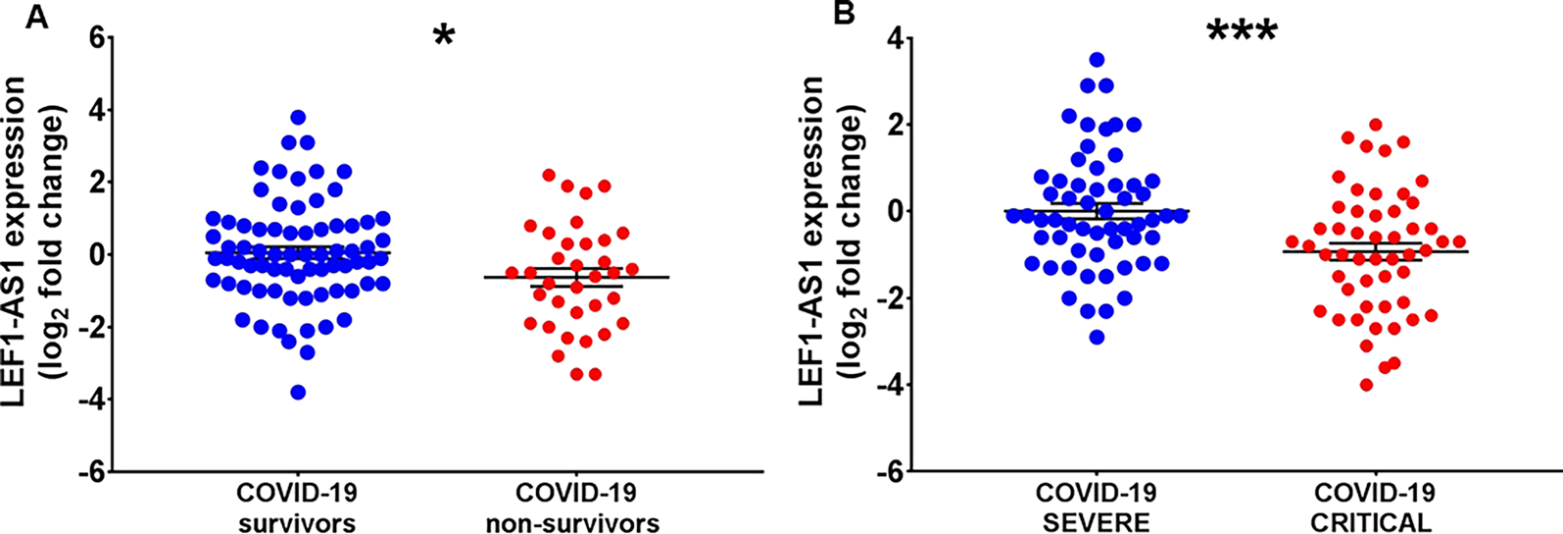
LEF1-AS1 expression is decreased according to COVID-19 mortality and severity in COVID-19 patients recruited at PSD. Total RNA was extracted from PBMC derived from non-surviving (n = 35) and surviving (n = 73) (**A**), or from critical (n = 55) and severe (n = 56) (**B**) COVID-19 patients. Dot-plots show lncRNAs relative expression measured by RT-qPCR and expressed as log_2_ fold change. Mean values and standard error bars are indicated. Mann–Whitney t-test: *p < 0.05, *** p < 0.001

**Fig. 4:**
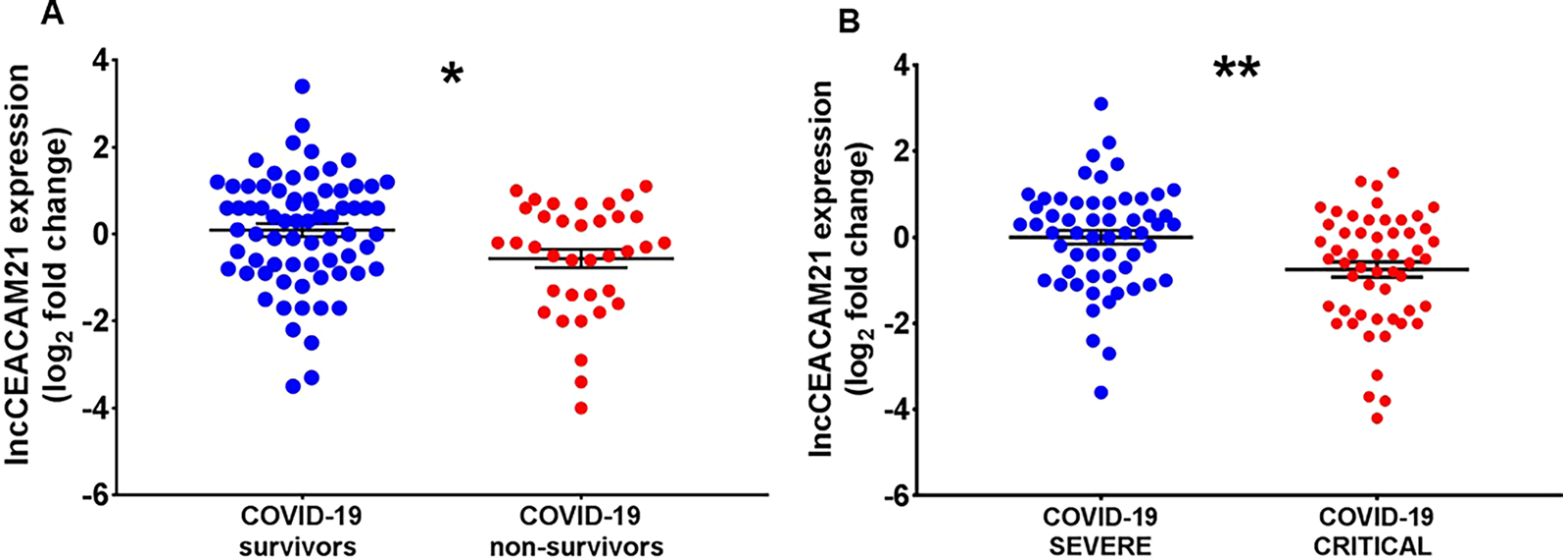
Decreased lncCEACAM21 expression in non-surviving and critical COVID-19 patients recruited at PSD. Total RNA was extracted from PBMC derived from non-surviving (n = 35) and surviving (n = 73) (**A**), or from critical (n = 55) and severe (n = 56) (**B**) COVID-19 patients. Dot-plots show lncRNAs relative expression measured by RT-qPCR and expressed as log_2_ fold change. Mean values and standard error bars are indicated. Mann–Whitney t-test: *p < 0.05, **p < 0.01

Also LEF1-AS1 (Fig. 3) and lncCEACAM21 (Fig. 4) displayed lower levels in non-survivors and critical patients, validating the results obtained in the profiling.

Odds ratios and AUCs calculations using a logistic regression model for all the HCG18 isoforms as well as LEF1-AS1 and lncCEACAM21 displayed statistically significant values comparing both critical vs severe patients and survivors vs non-survivors. Multivariate analysis showed that these lncRNAs were still significantly (p < 0.05) associated with the outcomes after age and sex-adjustment, in keeping with the lower age of the survivors in the validation groups (AUC values 0.74 for HCG18-242 and -244; Additional file 1: Table S5).

Additionally, neutrophil-to-lymphocyte ratios (NLRs) were calculated and they displayed a moderate inverse correlation to the expression of the mono-and multi-exonic HCG18 isoforms, of LEF1-AS1 and of lncCEACAM21 (Additional file 6: Figure S5).

**Fig. 5:**
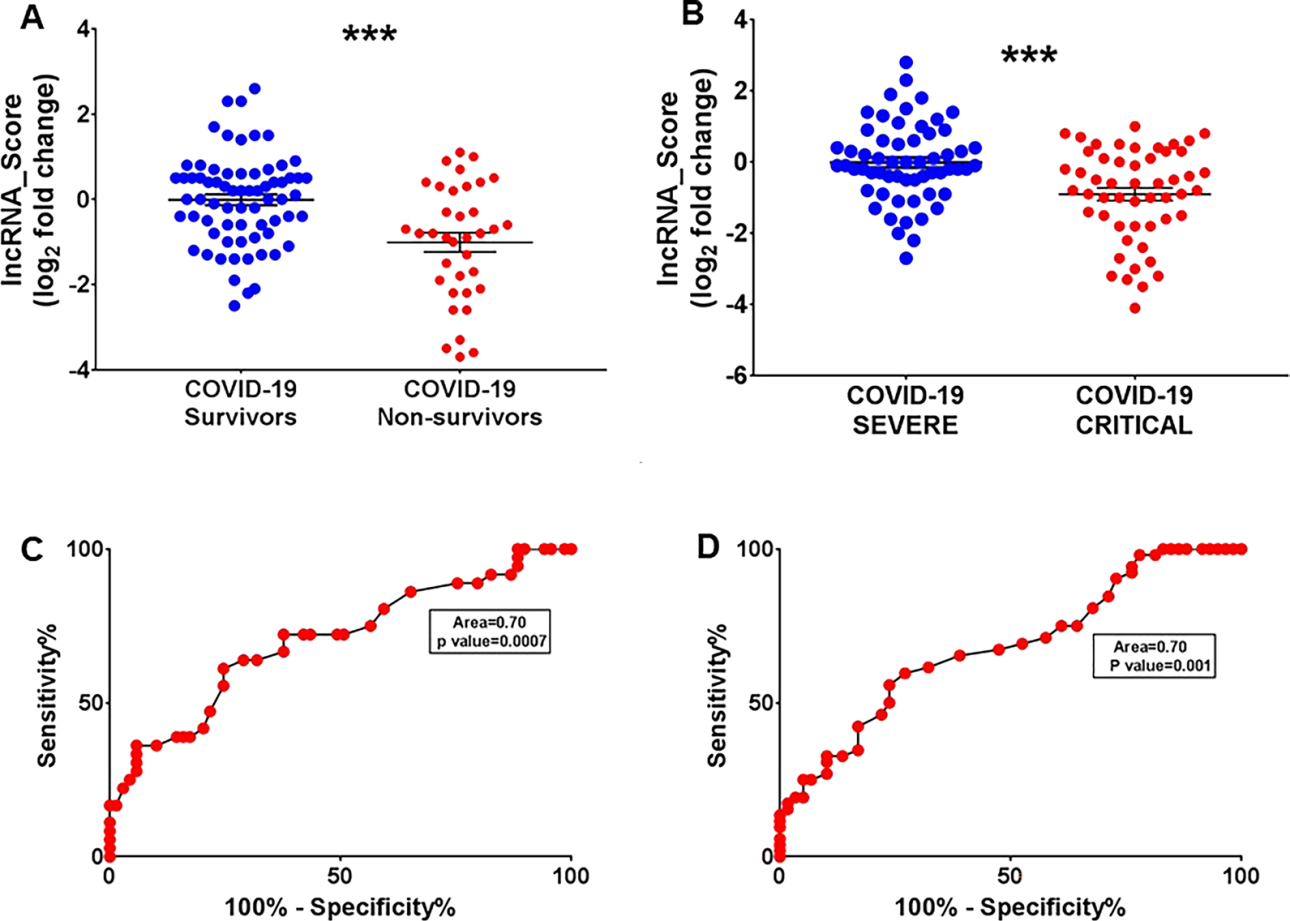
COVID19-LncRNAs score values segregate PSD COVID-19 patients according to mortality and severity. Total RNA was extracted from PBMC derived from non-surviving (n = 35) and surviving (n = 73), or from critical (n = 55) and severe (n = 56) COVID-19 patients. (**A**) and (**B**) Dot plots of log_2_ fold change averaged data from all COVID-19-LncRNAs (score) were compared between groups. Mean values and standard error bars are indicated. Mann–Whitney t-test: ***p < 0.001. (**C**)and (**D**) ROC curves for mortality and severity discrimination according to COVID-19-LncRNAs score mean values

We also tested the aggregated potential of the COVID19-lncRNAs, calculating a “lncRNA-score” by averaging the log_2_ fold change values for all COVID19-lncRNAs. As expected, COVID19-lncRNA-score values were significantly different for survivors and non-survivors (p < 0.0001, Fig. 5A) and for severe and critical (p < 0.0001, Fig. 5B) COVID-19 patients. By analyzing the ROC curve, the AUCs indicate a good discrimination between groups (Fig. 5C, D).

### Increased LEF1-AS1 levels in the plasma of critical and non-surviving COVID-19 patients

LncRNAs are also detectable in plasma [8]. Thus, the plasma levels of the COVID19-lncRNAs were measured in a subset of 77 PSD COVID-19 patients for whom a platelet-poor plasma sample was available. We observed that only LEF1-AS1 was readily measurable in plasma samples and its expression levels increased according to mortality and disease severity (Additional file 7: Figure S6), displaying a regulation opposite to that observed in PBMCs.

### Validation of deregulated lncRNAs in an independent cohort of COVID-19 patients

**Fig. 6:**
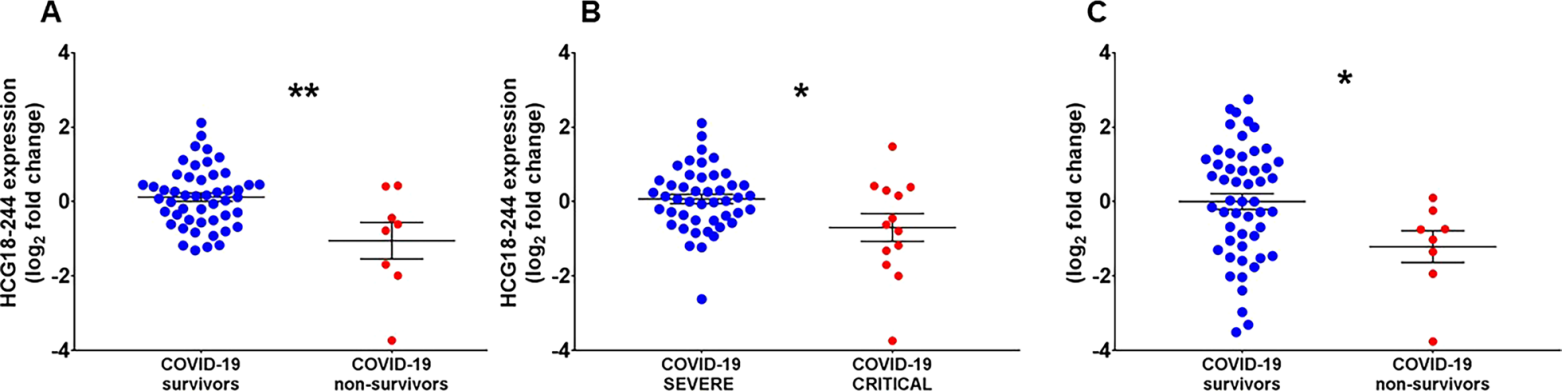
The expression levels of HCG18-244 and lncCEACAM21 are lower in non-surviving vs surviving and in critical vs severe COVID-19 patients recruited at MM. Total RNA was extracted from PBMC derived from non-surviving (n = 8) and surviving (n = 52) (**A** and **C**), or from critical (n = 13) and severe (n = 47) (**B**) COVID-19 patients. Dot-plots show lncRNAs relative expression measured by RT-qPCR and expressed as log_2_ fold change. Mean values and standard error bars are indicated. Mann–Whitney t-test: *p < 0.05, **p < 0.01

Next, we extended the validation of the COVID19-lncRNAs to an independent COVID-19 patient cohort. For this purpose, the peripheral blood was collected from 60 consecutive patients, positively tested for SARS-CoV-2 and hospitalized at IRCCS Multimedica (MM) for COVID-19 from March to May 2021. Like the PSD cohort, the critical patient group included ICU intubated or non-surviving subjects (n = 13), while the severe group included hospitalized surviving patients that were not subject to intubation (n = 47); as expected for consecutive patients, critical and non-surviving patients were significantly older compared to severe and surviving patients, respectively [27, 30] (Additional file 1: Table S6). The expression levels of the COVID19-lncRNAs were measured in the PBMCs by RT-qPCR, using the same experimental conditions used for PSD patients. In spite of the lower numerosity and disease severity in the MM cohort compared to the PSD one, we observed decreased levels of HCG18-244 in non-surviving (Fig. 6A) and critical (Fig. 6B) patients and of lncCEACAM21 in non-surviving patients (Fig. 6C) compared to relevant controls.

### Cell type expression of COVID-19-lncRNAs

To assess the tissue and cell expression pattern of COVID19-lncRNAs in COVID-19 patients, we took advantage of publicly available single-cell transcriptomics datasets of peripheral blood [22] as well as of post-mortem samples of heart, lung, kidney and liver [23] of COVID-19 infected patients. Only HCG18 was readily detectable, although not isoform level information could be extracted.

In the blood (Additional file 8: Figure S7A), HCG18 was readily measurable in B, CD4+ and CD8+ T lymphocytes and in plasmacytoid dendritic cells (DC), but also in monocytes and NKTs. In all of these cell types, lower expression levels were observed in patients affected from a severe/critical disease form.

At the tissue level (Additional file 8: Figure S7B), HCG18 was readily detectable in heart and kidney tissues from COVID-19-affected patients; cardiomyocyte and vascular endothelial cells expressed moderate levels of HCG18.

**Fig. 7:**
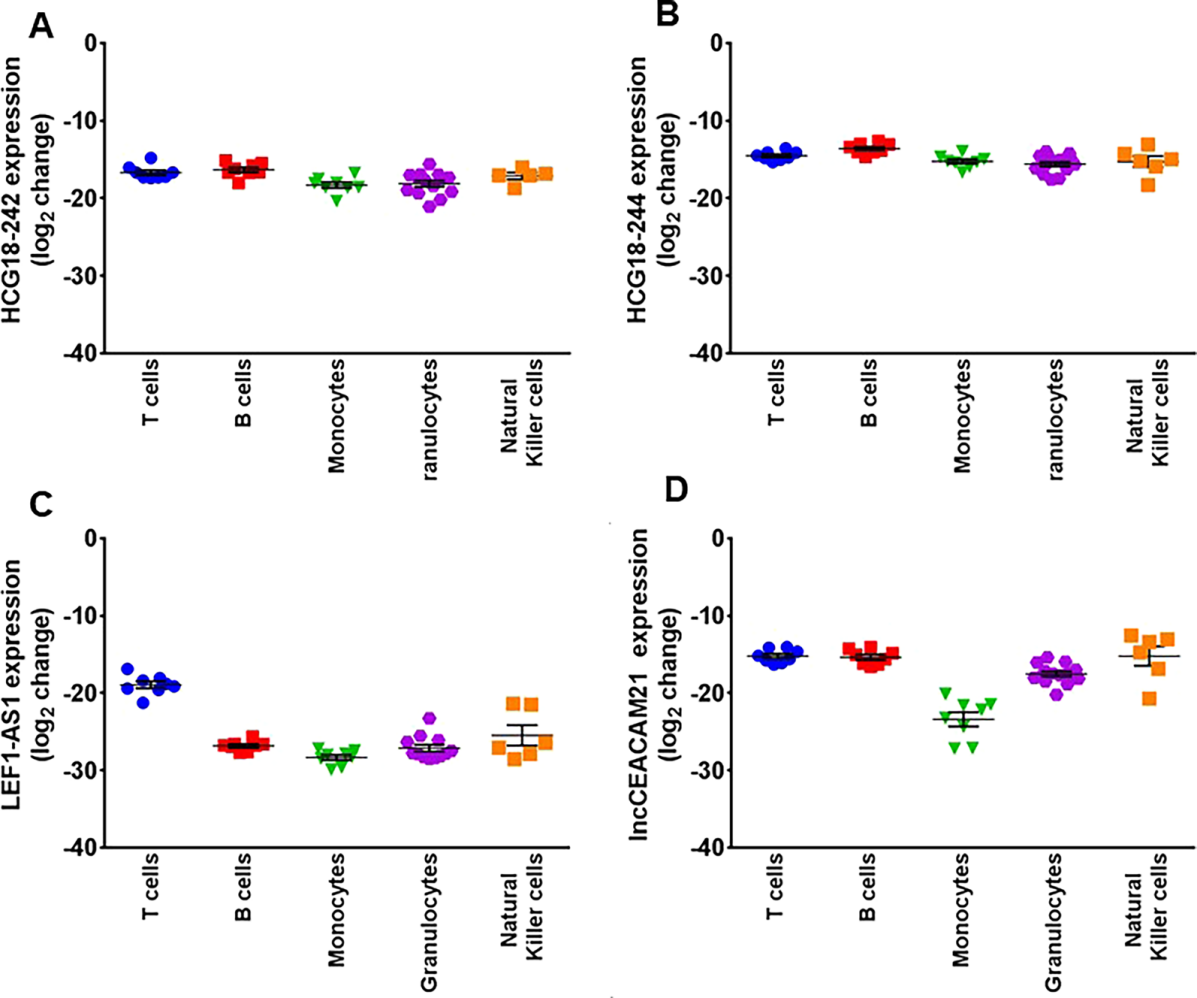
HCG18-242 (**A**), HCG18-244 (**B**), LEF1-AS1 (**C**) and lncCEACAM21 (**D**) expression in T- and B-lymphocytes, monocytes, neutrophils and natural killer cells (NK) in donor healthy patients. Dot-plot of average of inverted dCT values measured by RT-qPCR in the indicated cell populations. 18S levels were used for normalization. Mean values and standard error bars are indicated

To corroborate the data obtained by the single-cell transcriptomics of the peripheral blood, the expression of HCG18-242 and -244, LEF1-AS1 and lncCEACAM21 was measured in the main PBMC populations and neutrophils. To this aim, B and T lymphocytes, monocytes, granulocytes and NKTs were isolated by FACS-sorting from the blood of healthy donors and total RNA was extracted. HCG18-242 and -244, lncCEACAM21 were well expressed in all of the isolated cell subpopulations (Fig. 7A, B, D), while LEF1-AS1 was better detectable in T-lymphocytes and in NKTs (Fig. 7C).

Finally, the nucleo-cytoplasmic distribution of COVID19-lncRNAs was investigated. RNA was extracted from Jurkat cell fractions and the effectiveness of the fractionation procedure was monitored by measuring the lncRNA NEAT-1 mRNA and the 18S rRNA, which have a well-known nuclear and cytoplasmic distribution, respectively. Additional file 9: Figure S8 shows that COVID19-lncRNAs were expressed preferentially in the nucleus.

### Hypoxia modulates COVID19-lncRNAs expression in THP-1 monocytes

**Fig. 8:**
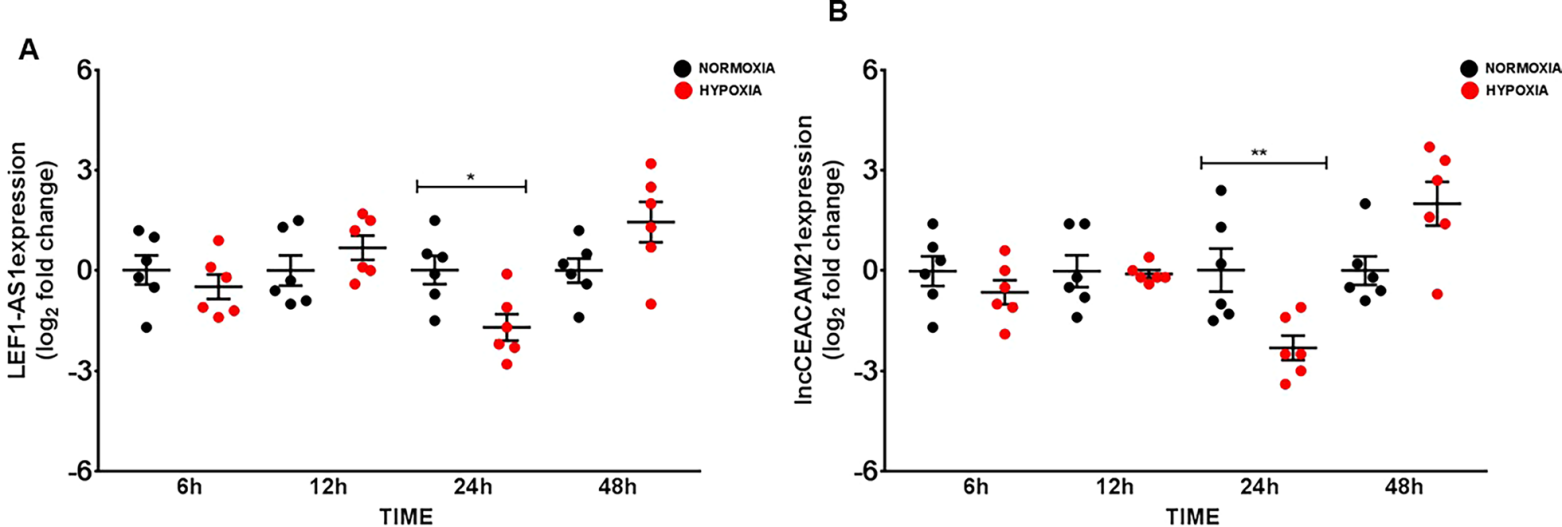
Lower LEF1-AS1 (**A**) and lncCEACAM21 (**B**) expression in hypoxic THP-1 cells. THP-1 cells were cultured in a hypoxia cabinet at 1% O_2_ and compared to cells in normoxic conditions (21% O_2_). Dot-plots show lncRNA relative expression measured by RT-qPCR and expressed as log_2_ fold change. Mean values and standard error bars are indicated. Mann–Whitney t-test: * for p < 0.05, **p < 0.01

In SARS-CoV-2 infected patients, low blood-oxygen levels are often found without dyspnea or other signs of respiratory distress. Thus, hypoxia is a relevant disease-associated stressor that may contribute to the gene expression changes observed in the PBMCs. In this view, cultured human monocyte cells (THP-1) were exposed to normoxia or hypoxia for 6, 12, 24 and 48 h, and the expression levels of HCG18 isoforms, LEF1-AS1 and lncCEACAM21 were measured. This cell line was adopted as it expressed readily detectable levels of all COVID19-lncRNAs. In this timeframe, no significant changes in cell viability was observed (data not shown). HCG-18 isoforms expression did not change, while LEF1-AS1 and lncCEACAM21 levels decreased significantly at 24 h of hypoxia (Fig. 8).

## Discussion

Report from the fifteenth meeting of the International Health Regulations (IHR) Emergency Committee reported that the COVID-19 pandemic disease is no longer a public health emergency of international concern [31]. Nonetheless, SARS-CoV-2 is continuing to infect or re-infect millions of persons and thousands of them are still dying [31]. Recently, an increase in COVID-19 cases and deaths has also been observed in South-East Asia, Eastern Mediterranean, and Western Pacific regions [32]. Moreover, despite the decreased incidence of the disease, the identification of new biomarkers predictive of severity and a deeper knowledge of COVID-19 pathogenetic mechanisms are still necessary. In particular, this may also be relevant to understand the onset of the syndrome referred to as “long COVID-19” [1, 33, 34].

Targeted RNA-seq technologies were used to monitor the expression of lncRNAs in PBMCs in relation to COVID-19 disease severity and outcomes. After a first round of technical validation and a second round of validation in a larger patient group, four lncRNAs resulted significantly associated with disease severity grade and outcome. The expression changes of COVID-19-lncRNAs were also assessed in an independent group of patients (MM group). Of note, the latter group was formed by patients enrolled in March–May 2021, a period corresponding to the third wave of COVID-19 pandemics in Europe, while PSD patients were enrolled during the first two waves. The third COVID-19 wave was characterized by a decrease in comorbidity burden, mortality, ICU admissions and invasive ventilation treatment compared to the first ones [35, 36]. These disease pattern may be also explained by the larger number of vaccinated individuals compared to the other ones [35]. Indeed, MM patients showed a lower mortality and ICU admission rate compared to those from PSD groups. The expression levels of HCG18-244 were confirmed to be significantly lower in critical and in non-surviving compared to severe and surviving patients, respectively, and lncCEACAM21 to be significantly decreased according to disease severity. These results indicate that HCG18-244 and lncCEACAM21 retain predictive sensitivity even when severity and mortality decrease. At the same time, together with patients characteristics, differences in the experimental strategy (consecutive vs matched patient comparison) may explain the different rates of validation between groups.

Lymphopenia and neutrophilia are hallmarks of several viral infectious diseases including SARS-CoV-2 infection [37–39] and these alterations were also observed in hospitalized COVID-19 patients recruited at both PSD and MM. Specifically, the neutrophil-to-lymphocyte ratio (NLR) is a biomarker of disease severity and mortality in COVID-19 patients [40–42]. We observed that the levels of the COVID19-lncRNAs were inversely correlated with NLR, suggesting that their down-regulation may be associated with the inflammatory status.

In platelet-poor plasma samples from PSD COVID-19 patients, we found that LEF1-AS1 was the only readily detectable COVID-19-lncRNAs and it was up- instead of down-regulated according to severity and outcome, as found in PBMCs. Among the possible causes underpinning lymphopenia during virus infections, there are several cell death-mediating mechanisms that promote the release of the cellular content [43]. Another reported cause of lymphopenia is the tissue re-distribution of lymphocytes, such as lymphocyte sequestration in the lungs [44, 45]. Thus, it may be hypothesized that the opposite expression pattern of LEF1-AS1 in plasma compared to PBMCs may be due to the release of the lncRNAs after lymphocytes or neutrophils lysis of cell compartments, or to the cell-to-cell communication mediated-trafficking of extracellular vesicles and exosomes [46–48]. In line with these data, it is possible to speculate that LEF1-AS1 can serve as COVID-19 severity and outcome predictor at two levels, one in the PBMCs and the other in the plasma, where it decreases or increases, respectively, at disease condition worsening.

LncRNA HCG18 (HLA Complex Group 18) participates in the biological development of several types of cancers [49]. In most cancers, HCG18 is up-regulated and exerts its oncogenic effects by regulating cancer cell proliferation, migration, invasion and apoptosis. However, in a small set of tumors it is down-regulated [50] and in this setting it may act as a tumor suppressor factor, indicating a highly context-dependent role. In the PBMCs of COVID-19 patients, we found that the expression of all three HCG18 transcripts decreased according to disease severity and outcome. In particular, HCG18 isoforms are annotated as antisense transcripts extending from HLA-L to TRIM39 genes. Major HLA-L belongs to the Histocompatibility Complex, Class I, which, together with genes of Class II, has been found significantly down-regulated in critical COVID-19 patients [51]. In keeping with these data and with the observed HCG18 down-regulation, it is possible to speculate that the transcription of the entire locus could be repressed during COVID-19.

LEF1-AS1 is up-regulated in many diseases, such as cell lung cancer and esophageal squamous cell carcinoma, and promotes cell proliferation and migration [52–55]. LEF1-AS1 is a transcript annotated on the opposite strand of the coding gene LEF1/TCF1 (lymphoid enhancer factor/ T-cell factor) and induces the expression of LEF1 mRNA epigenetically, by regulating H3K4 trimethylation in LEF1 promoter region [56]. LEF1, in turn, directly binds to β-catenin and activates the transcription of WNT-related genes, promoting T cell proliferation and differentiation [57], and also regulating chronic inflammation [58, 59]. Based on these findings, reduced expression of LEF1-AS1 may be associated with a reduced LEF1/TCF1 transcription and T-cell dysregulation during SARS-CoV-2 infection.

Relevant data regarding the expression changes of COVID-19-lncRNAs in the peripheral blood of patients are very scarce. Some evidence confirming our results came from the consultation of datasets of published studies that were not focused on our COVID19-lncRNAs. Due to the low expression of lncRNAs in blood samples, not all the lncRNAs were detectable in each analyzed dataset. The first dataset derives from the transcriptomic analysis of whole blood samples harvested from 359 severe and 106 non-severe COVID-19 cases [60]. In these patients, HCG18 was down-regulated in severe (requiring ICU, intubation/ventilation or oxygen support) patients compared to non-severe ones (mild and asymptomatic patients). Similarly, in another study performed on a limited number of COVID-19 patients and controls, the expression of HCG18 was lower in the plasma of both mild and severe patients compared to the healthy controls [61]. While our data agree with the aforementioned studies, none of them have investigated HCG18 expression at isoform level. Moreover, in other studies, LEF1-AS1 [11, 62] and lncCEACAM21 [15, 62] have also been found to be significantly decreased with disease worsening. Taken together these results supported the repressed expression of HCG18, LEF1-AS1 and lncCEACAM21 observed in our cohorts according to disease severity.

In SARS-CoV2-infected patients, low blood-oxygen levels are often observed and severe cases can progress to acute respiratory distress syndrome (ARDS), leading to organ dysfunction and failure. We have modeled in vitro the low oxygen levels observed in the blood by growing under hypoxia THP-1 cells, that express all the COVID-19-lncRNAs. We found that LEF1-AS1 and lncCEACAM21 expression decreased under hypoxic condition. Interestingly, it has been shown that both blood cells from COVID-19 patients and SARS-CoV-2 infected cells, in normoxia condition, accumulate hypoxia-inducible factor 1α (HIF-1α), eliciting inflammatory responses such as induction of chemokines and secretion of cytokines [63, 64]. In this scenario, the combination of HIF-1α activation and LEF1-AS1 decrease may both contribute to the dysregulation of myeloid cells in COVID-19 patients.

Interestingly, the expression of LEF1-AS1 has been also found to decrease in the whole blood of patients with cardiac fibrosis after myocardial infarction [65]. In addition, very recently, LEF1-AS1 and lncCEACAM21 transcripts have been found differently modulated in heart tissue of ischemic compared to non-ischemic dilated end-stage cardiomyopathy [16]. LEF1-AS1 and lncCEACAM21 are expressed in immune cells and, in chronic heart failure, it is well known the activation of the immune system, with the production and release of pro-inflammatory cytokines [66, 67]. These data, in summary, suggest that heart remodeling and COVID-19 disease may share some common mechanisms related to LEF1-AS1 and lncCEACAM21 expression changes.

## Conclusion

In conclusion, COVID-19 severity impacts on the lncRNA profile of the peripheral blood. Measurement of the PBMC levels of the identified COVID19-lncRNAs emerges as a potential non-invasive tool for risk-based stratification and mortality prediction of COVID-19 patients. Further studies in larger patient groups, displaying a broader severity, age and ethnic distribution, along with functional experiments, will be necessary to further validate these findings.

### Supplementary Information

**Additional file 1: Table S1.** COVID-19 patient’s characteristics, FIMICS profiling, PBMCs. Recruited at PSD. **Table S2.** COVID-19 patient’s characteristics, technical validation, PBMCs. Recruited at PSD. **Table S3.** Odds ratio of lncRNAs related to death using logistic regression model (with and without age adjustment) and the AUC. **Table S4.** Transcripts annotation and primer sequences. **Table S5.** Univariate and multivariable analysis adjusted for age and sex with odds ratio of lncRNAs related to mortality and severity and the AUC. **Table S6.** COVID-19 patient’s characteristics, PBMCs. Recruited at MM.

**Additional file 2: Figure S1.** COVID-19-lncRNAs amplicon characterization. Total RNA was extracted from PBMC of COVID19 patients recruited at PSD and reverse transcriptase reactions were run in the presence (RT+) or in the absence (RT−) of the enzyme, followed by PCR amplification for 40 cycles to detect the indicated genes. (A) Representative 2% agarose gel electrophoresis of RT+ and RT− samples (n = 3–4). For all primer couples, a single band was observed in RT+ condition only, with the exception of HCG18 ex 2–4 primers that were designed to detect multiple isoforms. (B) Representative assessment of the amplicon dissociation properties by melting curves analysis of the RT+ reactions (n = 3).

**Additional file 3: Figure S2.** Gating strategies used for FACS-sorting. Representative dot plots show the gating strategy used to identify and isolate the cell populations of interest, by FACS Sorting: (A) Monocytes (CD45+/CD14+/CD3−/CD19−), T (CD45+/CD14−/CD19−/CD3+) and B-lymphocytes (CD45+/CD14−/CD3−/CD19+) and (B) NK cells (CD3−/CD56+). SSC-A: side scatter area, FSC-A: forward scatter area, SSC-H: side scatter height. (C) Representative dot plots show the gating strategy used to characterize neutrophils (CD45+/CD14−/CD15+/CD66b+) by multicolor flow cytometry. SSC-A: side scatter area, FSC-A: forward scatter area, SSC-H: side scatter height.

**Additional file 4: Figure S3.** RNA-Sequencing profile of PBMC in COVID-19 patients (A) Volcano plot of differential expressed (DE) lncRNAs between 12 non-surviving and 13 surviving COVID-19 patients. Blue dots represent DE lncRNAs with p < 0.05 and |1| log_2_ fold change as threshold. (B) Heatmap of top 10 COVID-19 DE lncRNAs. In the heatmap obtained by ClustVis (https://biit.cs.ut.ee/clustvis/) values are expressed as Pearson’s correlation coefficient. The unsupervised clustering showed a perfect segregation of non-surviving vs surviving COVID-19 patients.

**Additional file 5: Figure S4.** Decreased HCG18 long-isoforms expression according to COVID-19 mortality and severity in COVID-19 patients recruited at PSD. Total RNA was extracted from PBMC derived from non-surviving (n = 35) and surviving (n = 73) (A), or from critical (n = 55) and severe (n = 56) (B) COVID-19 patients. Dot-plots show lncRNAs relative expression measured by RT-qPCR and expressed as log_2_ fold change. Mean values and standard error bars are indicated. Mann–Whitney t-test: * p < 0.05, ** p < 0.01.

**Additional file 6: Figure S5.** Inverse correlation between COVID19-lncRNA expression in PBMC and neutrophil/lymphocyte ratio (NLR). The expression levels of HCG18 isoforms (A–C), LEF1-AS1 (D) and lncCEACAM21 (E) were measured by RT-qPCR in PBMC and fold change values were correlated with neutrophil/lymphocytes ratio values of 80 COVID-19 patients by using Spearman’s correlation test.

**Additional file 7: Figure S6.** LEF1-AS1 levels in the plasma are increased according to COVID-19 mortality and severity in PSD COVID-19 patients. Total RNA was extracted from platelet-poor plasma samples derived from non-surviving (n = 35) and surviving (n = 42) (A), or from critical (n = 49) and severe (n = 28) (B) COVID-19 patients. Dot-plots show the lncRNA relative values measured by RT-qPCR and expressed as log_2_ fold change. Mean values and standard error bars are indicated. Mann–Whitney t-test: ** p < 0.01, ***p > 0.001.

**Additional file 8: Figure S7.** Broad expression of HCG18 in cells and tissues analyzed by single cell RNA-sequencing. The expression of HCG18 was analyzed in single-cell transcriptomics datasets derived from the blood of healthy controls and mild/moderate and severe/critical COVID-19 patients (http://covid19.cancer-pku.cn/#/summary) (A), as well as from samples of heart (B) and lung (C) of COVID-19 infected patients (https://singlecell.broadinstitute.org/single_cell/study/SCP1052/covid-19-lung-autopsy-samples). Values are expressed as log CPM. B = B lymphocytes; CD4 = helper T lymphocytes; CD8 = cytotoxic T lymphocytes; DC = Dendritic cells; Epi = Epithelial cells; Macro = macrophages cells; Mega = Megakaryocytes; Mono = Monocyte cells; NK = natural killer cells; Plasma = plasma cells.

**Additional file 9: Figure S8.** Nuclear expression of COVID19-lncRNAs. The expression of HCG18-242/244 and LEF1-AS1 was measured by RT-qPCR in cytoplasm (CYT) and nuclear cell fractions of Jurkat cells. Data are expressed as percentage compared to the total. Mean values and standard error bars are indicated.

## Data Availability

LncRNAs profiling results will be available after publication on Gene Expression Omnibus, number GSE217799 (https://www.ncbi.nlm.nih.gov/geo/query/acc.cgi?acc=GSE217799).

## Abbreviations

COVID-19: Coronavirus Disease-19
DE: Differentially expressed
ICU: Intensive care unit
lncRNAs: Long noncoding RNAs
MM: IRCCS MultiMedica
NKTs: Natural killer T cells
PBMCs: Peripheral blood mononuclear cells
PSD: IRCCS Policlinico San Donato
SARS-CoV-2: Severe acute respiratory syndrome coronavirus 2
