# Supplementary figures and images for: HCG18, LEF1AS1 and lncCEACAM21 as biomarkers of disease severity in the peripheral blood mononuclear cells of COVID-19 patients
Source: J Transl Med. 2023 Oct 26;21:758. doi: 10.1186/s12967-023-04497-6 (PMC10605335; doi:10.1186/s12967-023-04497-6)

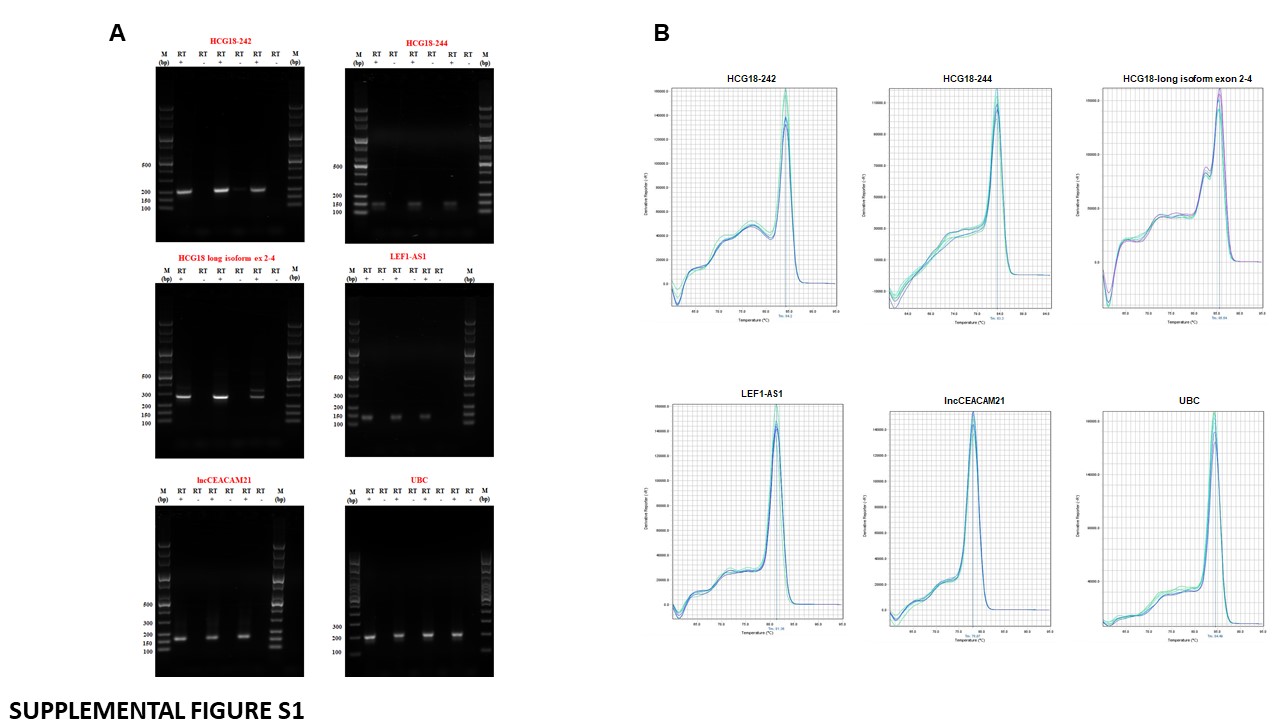

Supplement: Supplementary file 2 — Additional file 2: Figure S1. COVID-19-lncRNAs amplicon characterization. Total RNA was extracted from PBMC of COVID19 patients recruited at PSD and reverse transcriptase reactions were run in the presence (RT+) or in the absence (RT−) of the enzyme, followed by PCR amplification for 40 cycles to detect the indicated genes. (A) Representative 2% agarose gel electrophoresis of RT+ and RT− samples (n = 3–4). For all primer couples, a single band was observed in RT+ condition only, with the exception of HCG18 ex 2–4 primers that were designed to detect multiple isoforms. (B) Representative assessment of the amplicon dissociation properties by melting curves analysis of the RT+ reactions (n = 3). [file 12967_2023_4497_MOESM2_ESM.jpg]

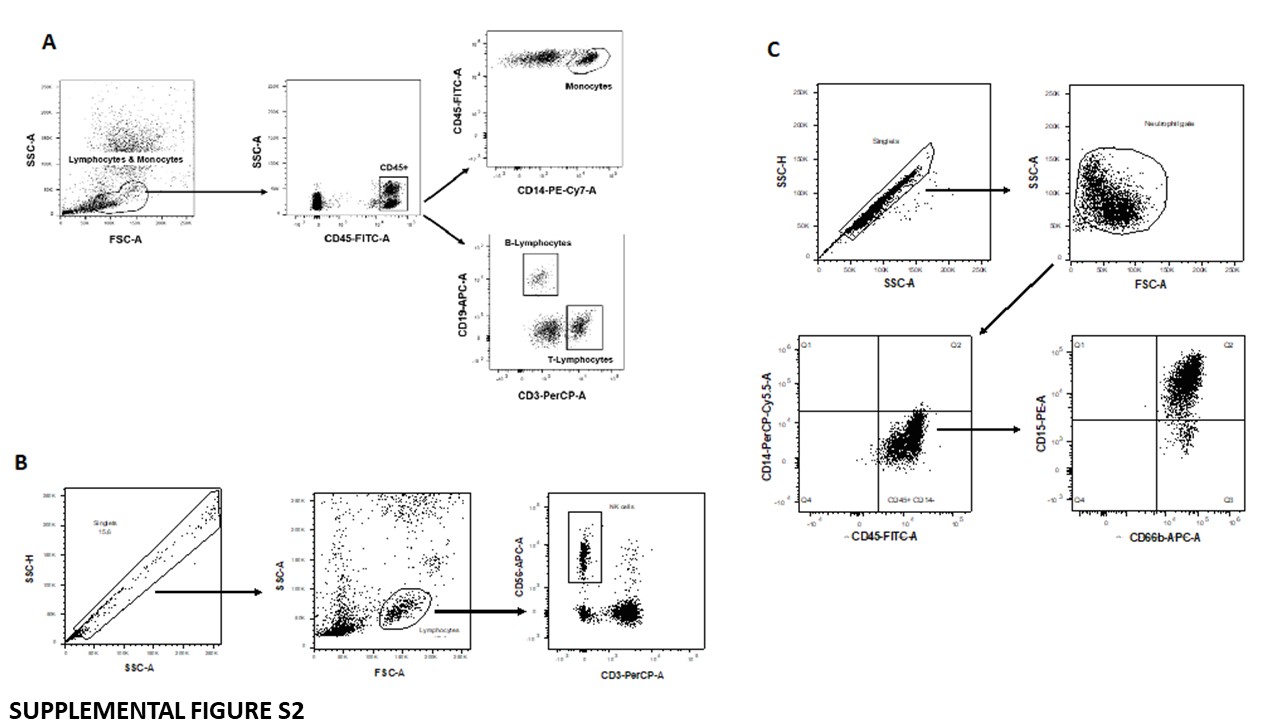

Supplement: Supplementary file 3 — Additional file 3: Figure S2. Gating strategies used for FACS-sorting. Representative dot plots show the gating strategy used to identify and isolate the cell populations of interest, by FACS Sorting: (A) Monocytes (CD45+/CD14+/CD3−/CD19−), T (CD45+/CD14−/CD19−/CD3+) and B-lymphocytes (CD45+/CD14−/CD3−/CD19+) and (B) NK cells (CD3−/CD56+). SSC-A: side scatter area, FSC-A: forward scatter area, SSC-H: side scatter height. (C) Representative dot plots show the gating strategy used to characterize neutrophils (CD45+/CD14−/CD15+/CD66b+) by multicolor flow cytometry. SSC-A: side scatter area, FSC-A: forward scatter area, SSC-H: side scatter height. [file 12967_2023_4497_MOESM3_ESM.jpg]

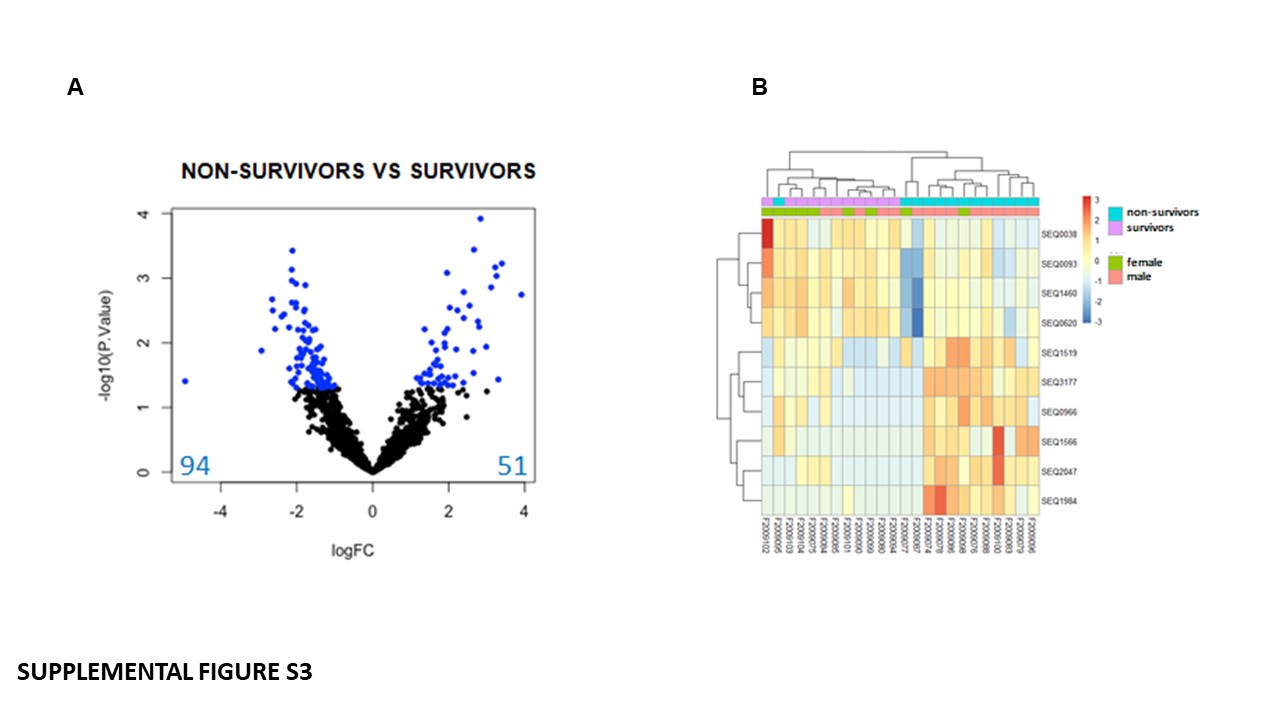

Supplement: Supplementary file 4 — Additional file 4: Figure S3. RNA-Sequencing profile of PBMC in COVID-19 patients (A) Volcano plot of differential expressed (DE) lncRNAs between 12 non-surviving and 13 surviving COVID-19 patients. Blue dots represent DE lncRNAs with p < 0.05 and |1| log2 fold change as threshold. (B) Heatmap of top 10 COVID-19 DE lncRNAs. In the heatmap obtained by ClustVis (https://biit.cs.ut.ee/clustvis/) values are expressed as Pearson’s correlation coefficient. The unsupervised clustering showed a perfect segregation of non-surviving vs surviving COVID-19 patients. [file 12967_2023_4497_MOESM4_ESM.jpg]

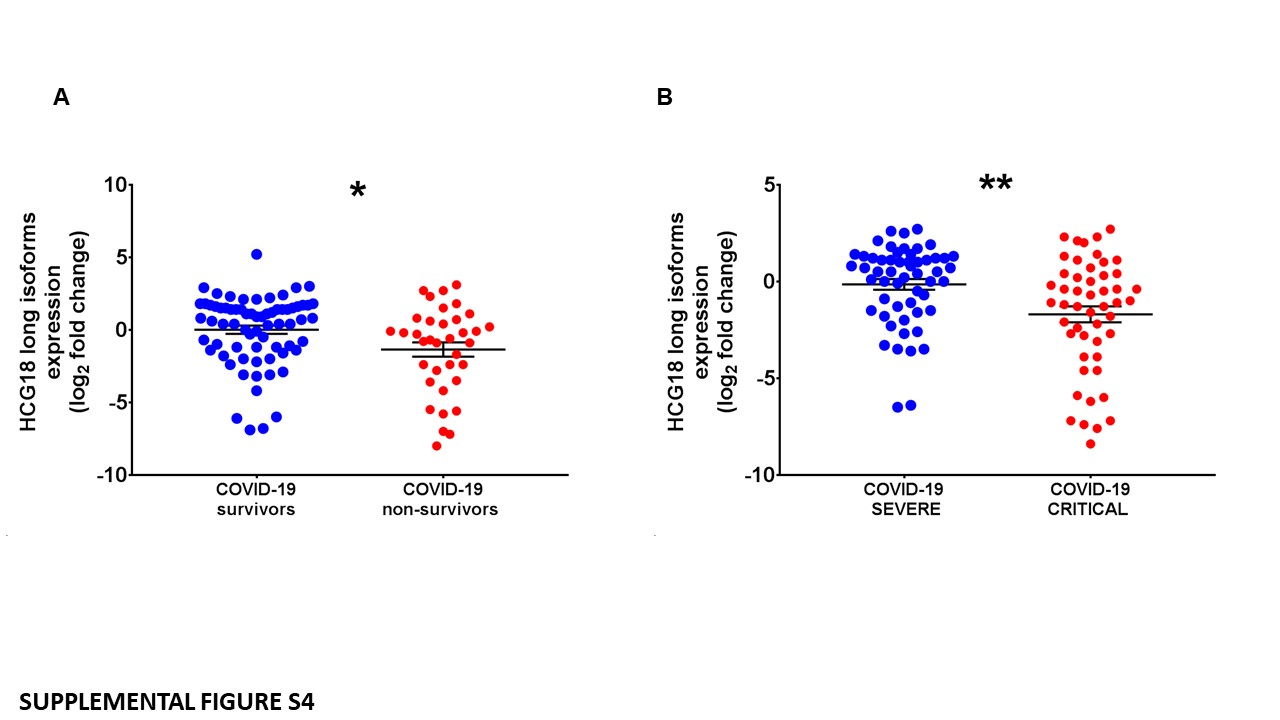

Supplement: Supplementary file 5 — Additional file 5: Figure S4. Decreased HCG18 long-isoforms expression according to COVID-19 mortality and severity in COVID-19 patients recruited at PSD. Total RNA was extracted from PBMC derived from non-surviving (n = 35) and surviving (n = 73) (A), or from critical (n = 55) and severe (n = 56) (B) COVID-19 patients. Dot-plots show lncRNAs relative expression measured by RT-qPCR and expressed as log2 fold change. Mean values and standard error bars are indicated. Mann–Whitney t-test: * p < 0.05, ** p < 0.01. [file 12967_2023_4497_MOESM5_ESM.jpg]

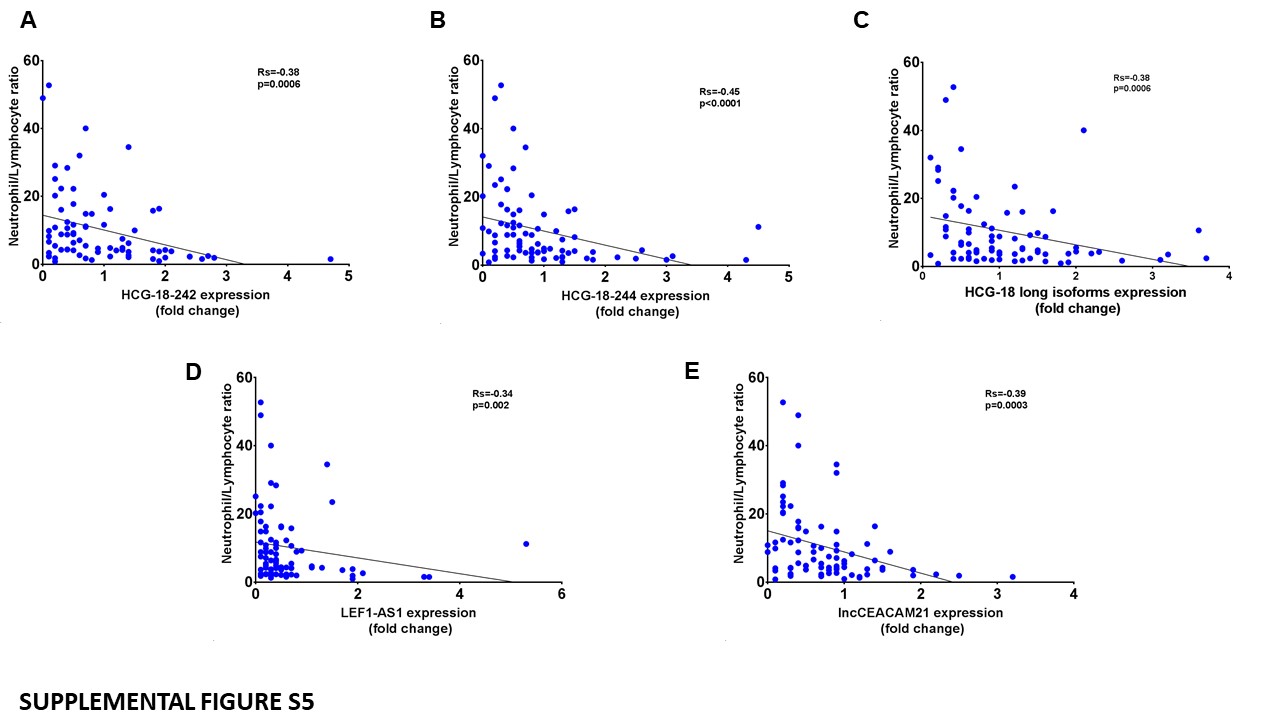

Supplement: Supplementary file 6 — Additional file 6: Figure S5. Inverse correlation between COVID19-lncRNA expression in PBMC and neutrophil/lymphocyte ratio (NLR). The expression levels of HCG18 isoforms (A–C), LEF1-AS1 (D) and lncCEACAM21 (E) were measured by RT-qPCR in PBMC and fold change values were correlated with neutrophil/lymphocytes ratio values of 80 COVID-19 patients by using Spearman’s correlation test. [file 12967_2023_4497_MOESM6_ESM.jpg]

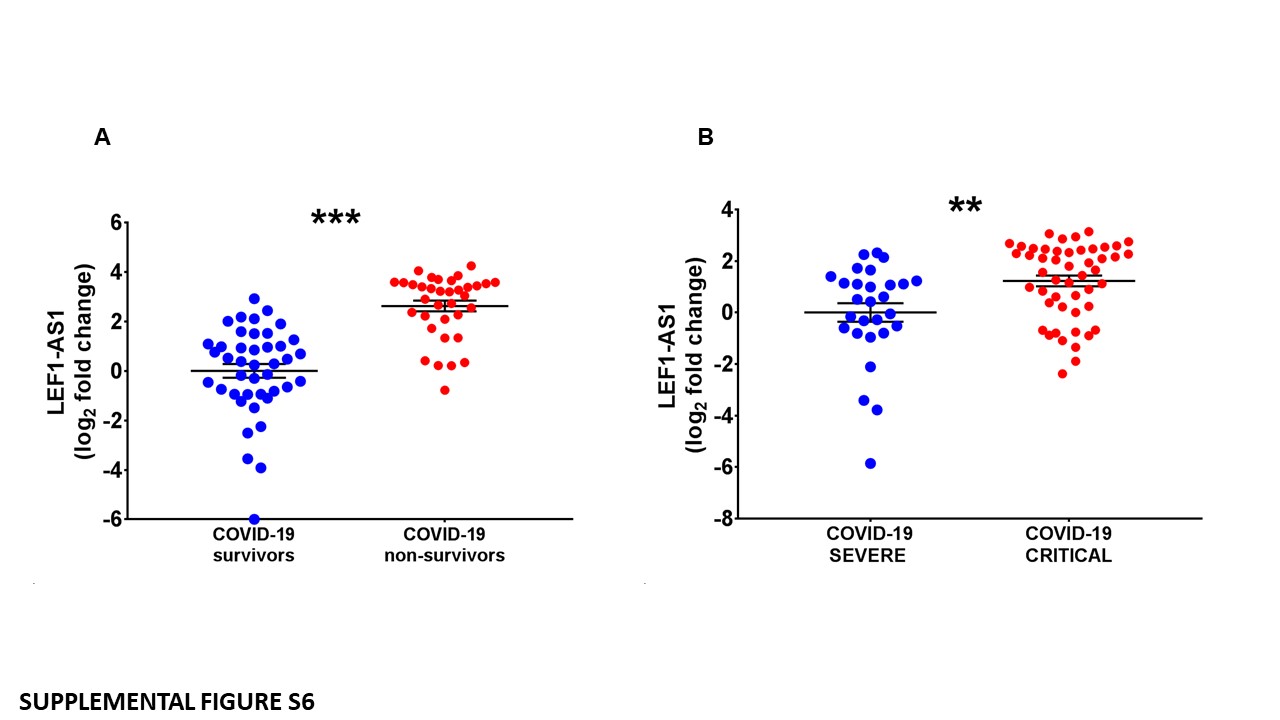

Supplement: Supplementary file 7 — Additional file 7: Figure S6. LEF1-AS1 levels in the plasma are increased according to COVID-19 mortality and severity in PSD COVID-19 patients. Total RNA was extracted from platelet-poor plasma samples derived from non-surviving (n = 35) and surviving (n = 42) (A), or from critical (n = 49) and severe (n = 28) (B) COVID-19 patients. Dot-plots show the lncRNA relative values measured by RT-qPCR and expressed as log2 fold change. Mean values and standard error bars are indicated. Mann–Whitney t-test: ** p < 0.01, ***p > 0.001. [file 12967_2023_4497_MOESM7_ESM.jpg]

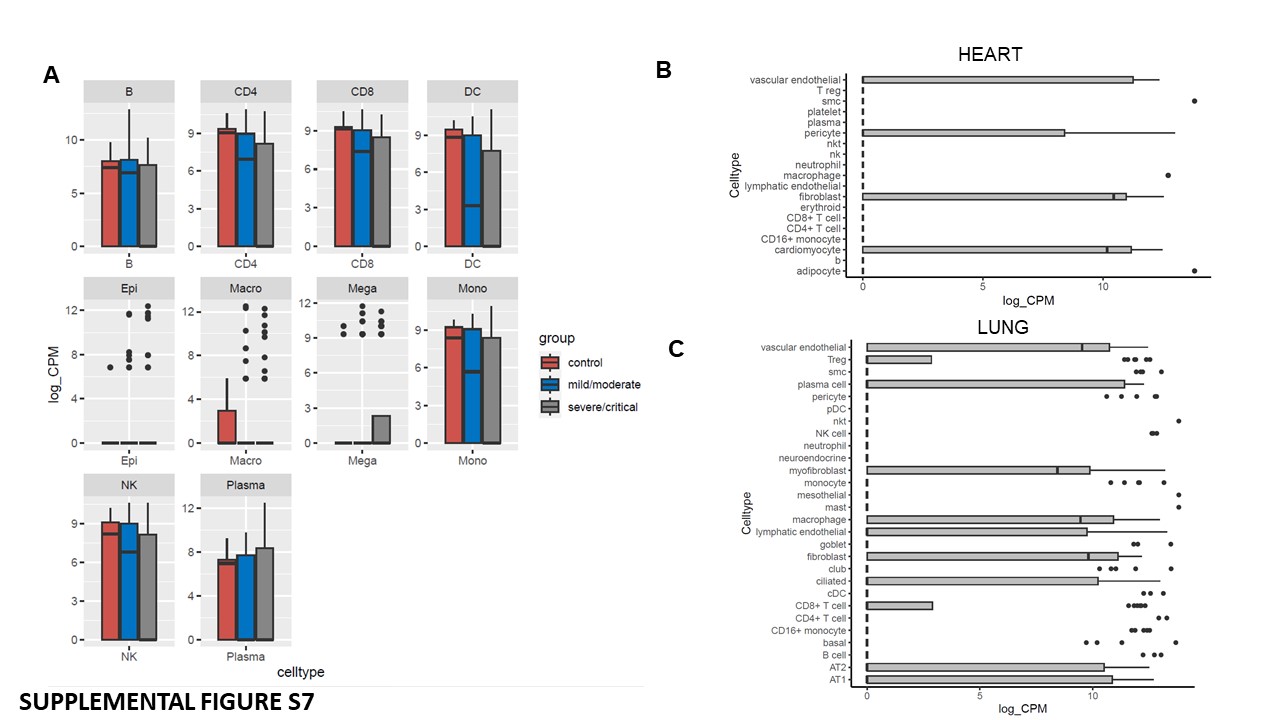

Supplement: Supplementary file 8 — Additional file 8: Figure S7. Broad expression of HCG18 in cells and tissues analyzed by single cell RNA-sequencing. The expression of HCG18 was analyzed in single-cell transcriptomics datasets derived from the blood of healthy controls and mild/moderate and severe/critical COVID-19 patients (http://covid19.cancer-pku.cn/#/summary) (A), as well as from samples of heart (B) and lung (C) of COVID-19 infected patients (https://singlecell.broadinstitute.org/single_cell/study/SCP1052/covid-19-lung-autopsy-samples). Values are expressed as log CPM. B = B lymphocytes; CD4 = helper T lymphocytes; CD8 = cytotoxic T lymphocytes; DC = Dendritic cells; Epi = Epithelial cells; Macro = macrophages cells; Mega = Megakaryocytes; Mono = Monocyte cells; NK = natural killer cells; Plasma = plasma cells. [file 12967_2023_4497_MOESM8_ESM.jpg]

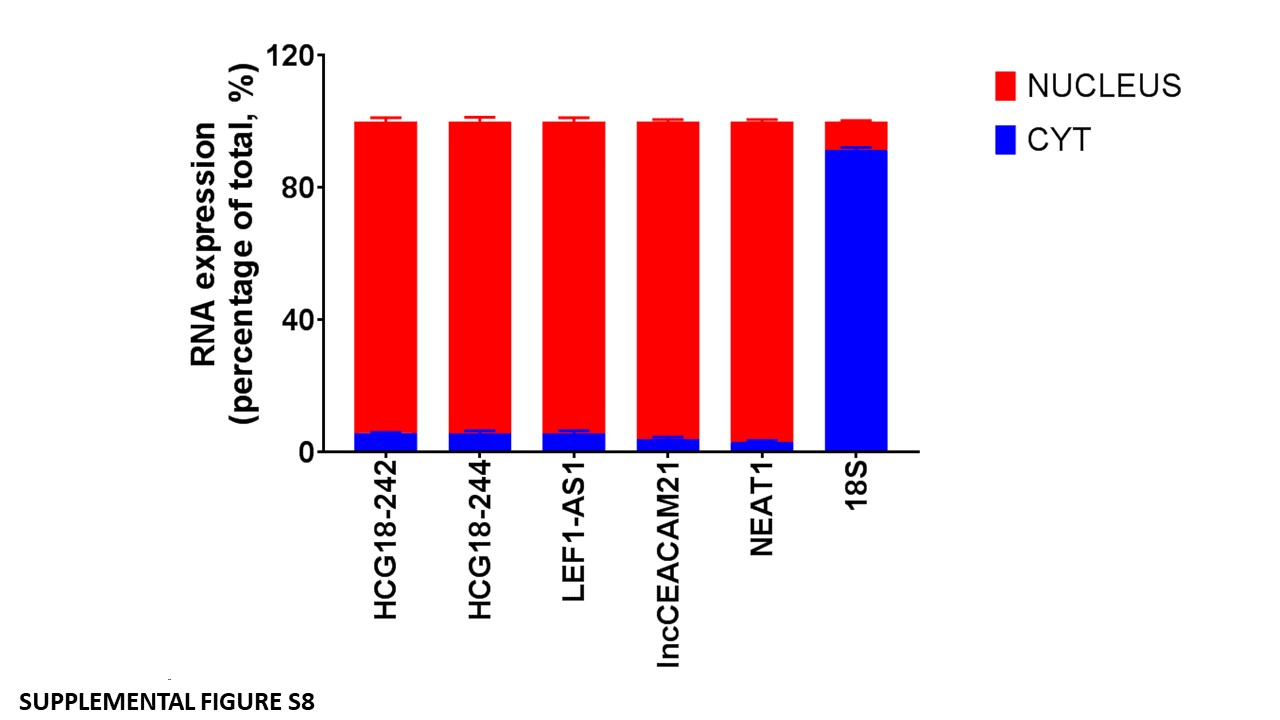

Supplement: Supplementary file 9 — Additional file 9: Figure S8. Nuclear expression of COVID19-lncRNAs. The expression of HCG18-242/244 and LEF1-AS1 was measured by RT-qPCR in cytoplasm (CYT) and nuclear cell fractions of Jurkat cells. Data are expressed as percentage compared to the total. Mean values and standard error bars are indicated. [file 12967_2023_4497_MOESM9_ESM.jpg]
